# Fabrication of spectroscopic microfluidic chips for mastitis detection in raw milk

**DOI:** 10.1038/s41598-023-33258-0

**Published:** 2023-04-13

**Authors:** Chalinee Phiphattanaphiphop, Komgrit Leksakul, Wasawat Nakkiew, Rungrueang Phatthanakun, Trisadee Khamlor

**Affiliations:** 1 Department of Industrial Engineering, Faculty of Engineering, Chiang Mai University, Chiang Mai, 50200 Thailand; 2 Department of Mechatronics Engineering, College of Integrated Science and Technology, Rajamangala University of Technology Lanna, Chiang Mai, 50220 Thailand; 3 Synchrotron Light Research Institute, Nakhon Ratchasima, 30000 Thailand; 4 Department of Animal and Aquatic Science, Faculty of Agriculture, Chiang Mai University, Chiang Mai, 50200 Thailand

**Keywords:** Engineering, Techniques and instrumentation

## Abstract

Mastitis is a disease that directly affects the quantity and quality of milk produced by dairy cows, which can have a negative impact on the income generated from selling the milk. Severe inflammation caused by this mammary disease can result in up to 1 × 10^6^ white blood cells per milliliter of cow milk. Currently, the California mastitis test is a popular chemical inspection test, but its error rate of over 40% is a significant factor in the ongoing spread of mastitis. In this study, a new microfluidic device was designed and fabricated to identify normal, sub-clinical, and clinical mastitis. This portable device allows for precise and analysis of results within a second. The device was designed to screen somatic cells and a staining process was added to identify somatic cells using single-cell process analysis. The fluorescence principle was used to identify the infection status of the milk, which was analyzed using a mini-spectrometer. The accuracy of the device was tested, and it was found to determine the infection status with 95% accuracy, compared to the accuracy obtained using the Fossomatic machine. By introducing this new microfluidic device, it is believed that the spread of mastitis in dairy cows can be significantly reduced, leading to higher quality and more profitable milk production.

## Introduction

Mastitis in dairy cows is an ongoing problem faced by dairy farmers as it directly affects the quantity and quality of raw milk, resulting in substantial economic losses. The global annual loss is estimated to be around **€**30 billion^1^, and is made up of significant milk losses, poor milk quality, the elimination of chronically infected animals, and occasional deaths^2^. Mastitis is an inflammation of the mammary glands caused by the invasion of certain pathogens, allergies, or physical trauma^3^, resulting in abnormal changes in the mammary glands and milk. There are generally two types of mastitis: clinical and subclinical mastitis. These occur due to various causes, including bacterial infections, infectious toxins wounds, or chemical exposure, with the most primary and frequent cause being bacterial infection. Mastitis can be transmitted from cattle to cattle, increasing its spread. If the symptoms of mastitis become apparent after a delay in detection, it can significantly impede the healing of the dairy cow for approximately 2 weeks. This can result in blindness and a permanent inability for the cow to produce milk. Additionally, routinely managing mastitis involves administering antibiotics to treat and prevent the disease, which poses a serious risk of antibiotic resistance in the cow. There are two stages of diagnosis: the first involves assessing the disease's status to determine its presence, and the second involves detecting the causative agent. For severe mastitis, the status of the disease can be judged by observing the breast’s appearance and milk. To screen for clinical or subclinical mastitis, on-farm screening tests for the analysis of the somatic cell count (SCC), such as the California mastitis test (CMT), are employed. Determining the SCC of milk involves evaluating the somatic cells (SCs) through laboratory microscopy, where the SCs of the milk are directly observed in microscopic slides as stained cells and counted using an operator. Analyzing the results using this method is time consuming and requires specialists to prepare the sample^4^. Flow cytometry (FCM) can be used for the relatively rapid SCC and bacterial analysis of milk^5^; this method also requires the support of skilled personnel to handle relatively expensive hardware needed for the analysis and other disposable items.

CMT reagents can be simply used for the mastitis test and applied for SCC estimation on a farm^5^. The reagent used in the CMT is sodium alkyl aryl sulfonate, based on the principle that nucleic acids and the cells of other elements are released in the presence of high SCC and gel formation, which is easily detectable^6,7^. However, the results obtained with this reagent are false positives or false negatives, and low sensitivities and specificities are observed for the SCC^6–8^. Neither of these tests provided an accurate numerical value for the SCC because the results were based on the morphological characteristics perceived by the test subject’s eye, thereby resulting in inaccuracies. Additionally, these tests do not identify the pathogen involved and the virulence of the infection but only provide positive or negative outcomes for mastitis^9,10^.

Currently, there are SCC detectors such as the Coulter counter (Coulter Electronics Ltd., Luton) and other automatic instruments that measure SCC indirectly, such as the Fossomatic and DeLaval cell counters (DeLaval, Tumba, Sweden). Coulter counter is a high-speed device used for particle size analysis that works on the principle of an electronic particle counter. Using this counter involves adding a formaldehyde solution to the milk to be examined to analyze the somatic cells and eliminate the fat particles by treating them with a lysing solution that overlaps in the size range of the cells^11^. Other automatic instruments that indirectly measure SCC are the Fossomatic 90, which works based on disk cytometry, and the Fossomatic 5000 (Foss Electric, Hillerød, Denmark) which is based on FCM. In FCM, a suspension of cells is stained and forced through a capillary tube illuminated in front of a microscope objective. Every passing cell is then registered by the photo-electronic device attached to the microscope. However, FCM-based machines are very expensive and can only be used in the laboratory. The DeLaval cell counter (DeLaval, Tumba, Sweden) is an analytical portable instrument for counting somatic cells optically and automatically. This method suffers from a high coefficient of variation and low repeatability^12^.

Nowadays, microfluidic technology can accurately analyze the results of mastitis status. Microfluidic devices are powerful tools for detecting and identifying biological particles from samples, especially pathogenic ones. Several microfluidic types have been proposed for clinical bacterial detection. For example, the blood and urine of a human were tested using a microfluidic device; the bacteria E.coli was separated from their red blood cells at high cell concentrations^13^. A microfluidic platform was used to identify multiple pathogenic bacteria that contributed to urinary tract infections; the accuracy of the results was at a rate greater than 90%^14,15^. Additionally, an automated dark field microscope was applied to simultaneously identify and quantify pathogenic bacteria. Live pathogenic bacteria were immobilized directly from the clinical samples in less than 4 h for both sample preparation and data collection^16^. These devices can also simultaneously measure the size of each bacterium^17^. Quite recently, two microfluidic platforms have been proposed for separating bacteria from blood cells at high cell concentrations using acoustophoresis in silicon^18^ and plastic microfluidic chips^19^, allowing the throughputs to be high enough for many clinical tasks^20^. However, these platforms rely on large hardware for managing flow control and they lack rapid detection capabilities.

Accordingly, we researched to develop a novel microfluidic device that allows for simple analysis of results based on a lab-on-a-chip approach. The microfluidic chip was designed with three parts: a filter, mixer, and detector, which enabled the one-way flow of the sample without any obstruction during testing. Somatic cells were filtered to achieve a size range of 10–30 μm and were then stained with acridine orange (AO). The somatic cells were aligned within a microchannel to enable precise control of cell positioning and orientation downstream at the detection area, which was integrated with fiber optics for fluorescence measurement. This device can be used to identify somatic cells in raw cow milk by measuring the fluorescence emitted by the cells when they are excited with a specific wavelength of light. The fluorescence signal can be used to distinguish somatic cells from other types of cells and identify the mastitis status in the milk. The results showed that the device can perform analysis with an accuracy of up to 95% compared to that of the Fossomatic machine.

## Materials and methods

This research classified the sample raw milk into three groups based on three experimental methods: CMT, Fossomatic, and the proposed method (mini-spectroscopy). The groups were labeled as normal, sub-clinical, and clinical by using the CMT and Fossomatic methods for comparison purposes. The comparison results were needed to demonstrate the effectiveness of the proposed method and provide a reference for the database.

The CMT results revealed five possible classes based on the gel formed by the mixture between the milk and the CMT reagent: 0 (negative), 1 (trace +), 2 (weak positive ++), 3 (distinct positive +++), and 4 (strong positive ++++). The Fossomatic method classified the milk based on the estimated somatic cell count (SCC), with milk below 200,000 cells/mL labeled as premium, milk between 200,000 and < 350,000 cells/mL labeled as good, and milk between 350,000 and 500,000 cells/mL labeled as standard^21^. The datasets were collected to interpret the results obtained with both the CMT and Fossomatic devices. However, high discrepancies were observed in the interpretation, particularly between degrees 1 and 2 and between degrees 3 and 4. As a result, a second group of labels was added to the sample. Samples classified as traces or weak positives were grouped under the label “sub-clinical”, samples classified as distinctive positive or strong positives were grouped under “clinical”, and samples classified as negative were grouped under “normal”. This classification was correlated with the number of somatic cells in the milk. Milk with a somatic cell count below 200,000 cells/mL was labeled as normal, milk with a count between 200,000 and 500,000 cells/mL was labeled as sub-clinical, and milk with a count greater than 500,000 cells/mL was labeled as clinical. Table 1 presents the instance distribution per class and interpretations. The results indicated that the prevalence of mastitis observed in this study was similar to that reported in previous researches in this area^22^.

**Table 1:** Interpretation of results under CMT and Fossomatic machine, and classification of milk according to results.

CMT	Interpretation	Fossomatic (cell/mL)	Class	Number of samples
0	Negative	< 200,000	Normal	40
+	Trace	200,000–350,000	Sub-clinical	40
++	Weak positive	350,000–500,000		
+++	Distinct positive	500,000–1,000,000	Clinical	20
++++	Strong positive	> 1,000,000		

### Preparation of standard substance and raw milk for the proposed method

**Figure 1:**
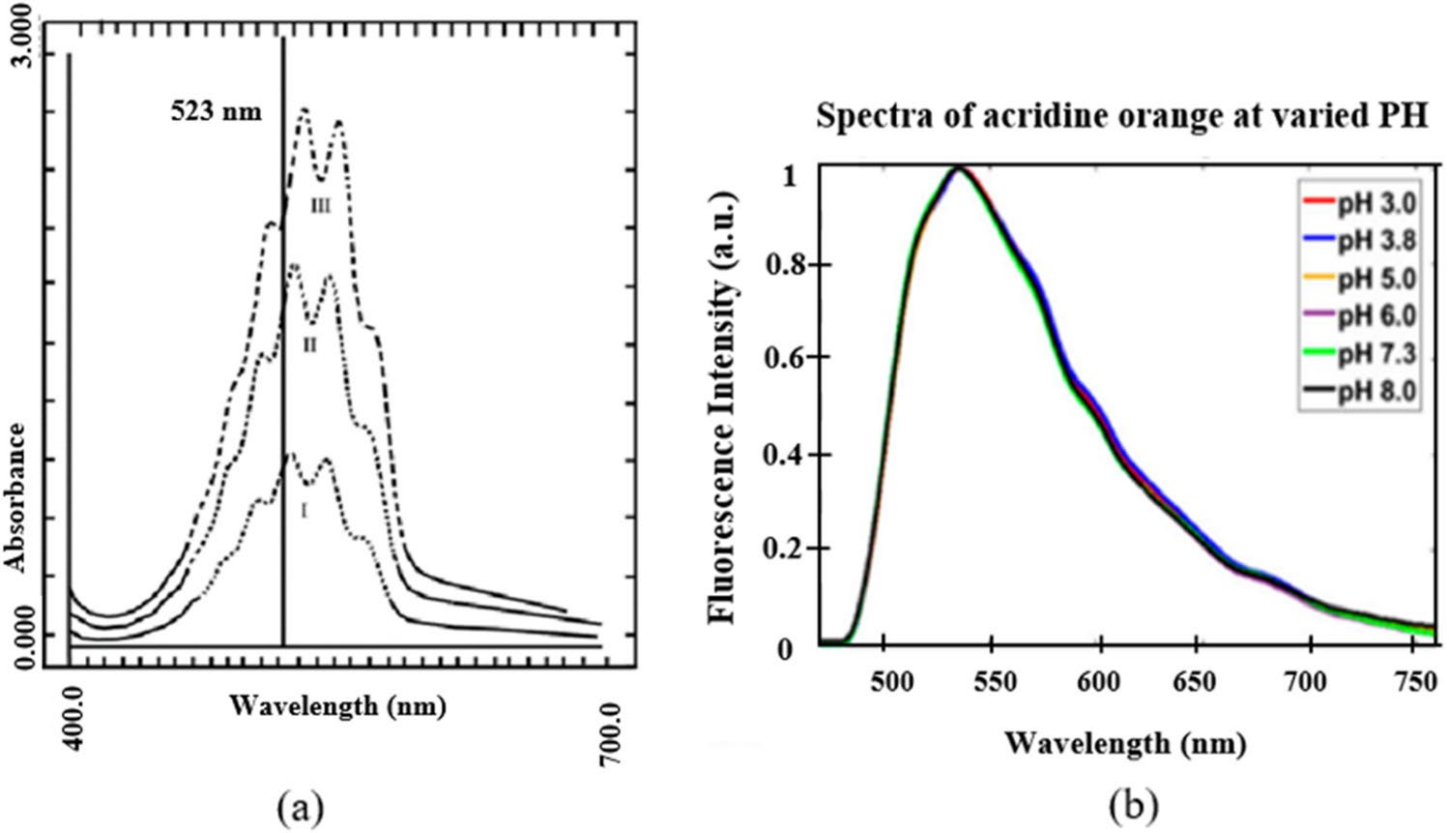
Emission spectra of substances: (**a**) standard potassium permanganate solution and (**b**) standard AO solution.

The microfluidic device and cuvette method were evaluated using potassium permanganate (KMnO_4_) as the standard substance for comparison. The device ability to analyze milk was tested in absorbance, transmittance, and fluorescence modes, with AO dye used in the fluorescence test. Figure 1a displays the maximum absorbance of KMnO_4_ during a time slice, along with its positive and negative deviation from the mean average absorbance at 523/10 nm^23^. Figure 1b shows the fluorescence emission spectra of an AO-labeled cell, which was measured using a spectrometer to record the spectrum of emitted light at 525/50 nm^24^. A KMnO_4_ solution was prepared at a concentration of 10–45 mg/dm^3^, and solutions of 100 µg/mL AO hemi (zinc chloride) salt (A6014, Sigma-Aldrich) were also prepared.

Raw milk samples were collected from 5 Holstein cows located in North Thailand. A total of 100 mL of raw milk was obtained in 1 day and stored in a refrigerator at less than 4 °C for 1 day after milking to preserve its quality. The samples were removed from the refrigerator 60 min prior to measurement and allowed to reach a temperature of 37 °C. To ensure that the components of the samples were uniform, they were stirred at 40 °C in a water bath^25^.

### Spectral collection system

**Figure 2:**
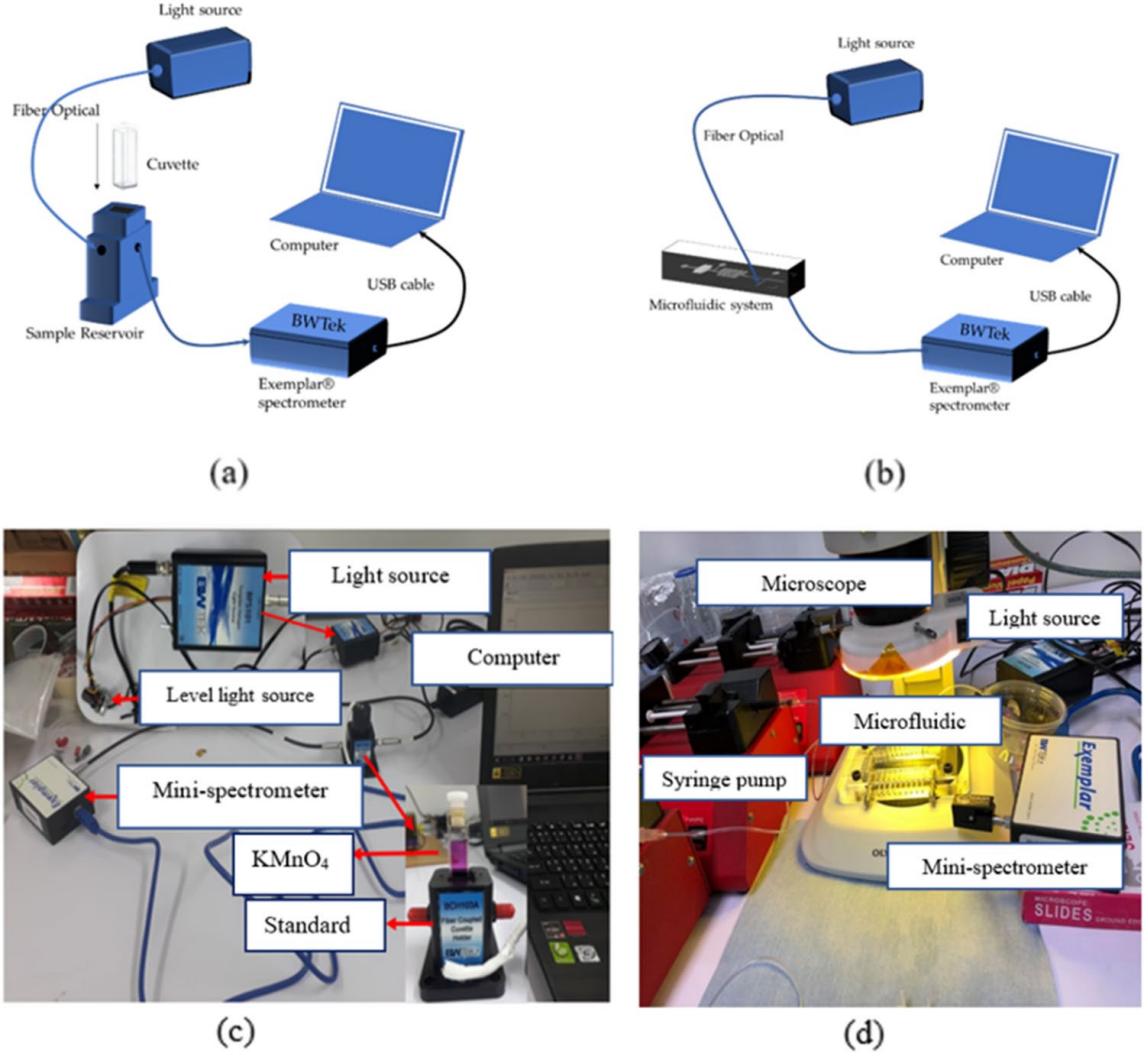
System used to obtain the fluorescence spectra. Analysis of milk under (**a**) standard system (**b**) microfluidic chip (**c**) experimental setup for sorting somatic cells under standard device (cuvette) and (**d**) experimental setup for sorting somatic cells under microfluidic device.

Figure 2 illustrates the spectroscopy system employed to acquire the fluorescence spectra. The system comprised an Exemplar spectrometer (B&W Tek, Inc., USA) with a wavelength range of 350–1050 nm, a quartz cuvette with an optical path of 2 mm serving as a sample pool (Yehui Instrument Ltd. Co., China), a sample reservoir (BCH103A B&W Tek, Inc., USA), a light source (BPS101 B&W Tek, Inc., USA), and 100 µm optical fibers (Ocean Optics, Inc., FL). The test standard was run on a laptop computer (Fig. 1a), whereas a microfluidic system replaced the quartz cuvette (Fig. 1b).

Optical fibers were used to connect the light source, sample reservoir, and spectrometer. Raw milk samples were prepared by mixing them with AO at a ratio of 10:1 and injecting them into quartz cuvettes using micropipettes. The cuvettes were filled with the diluted milk and placed in the sample reservoir. Fluorescence spectra of the samples were collected over a wavelength range of 350–1050 nm using the BWSpec software, with an integral time of 300 ms, an average number of 5, and a smoothing number of 0. Five replicates were created for each sample, and the mean value was calculated as the result of the standard system.

For the analysis of raw milk using the microfluidic chip system, the light source, microfluidic system, and spectrometer were connected using optical fibers. The raw milk was injected into the microfluidic system at a flow rate of 100 µL/min using a syringe pump, and the spectra were collected using the BWSpec software, with the same integral time, average number, and smoothing number as for the standard system. Five replicates were created for each sample, and the mean value was calculated as the result of the microfluidic system.

To ensure high-quality spectral data and minimize the impact of environmental noise and power consumption, the system was stabilized by powering it on for approximately 30 min before each experiment. During this period, the instrument was allowed to reach its operating temperature. To provide a constant current and high efficiency from the tungsten halogen lamp, the BPS101 Tungsten halogen light source that required a warm up period was used for the mini-spectrometer measurement. Therefore, it was necessary to allow sufficient warm-up time for the lamp to achieve a stable value of around 20 lumens per watt, which is comparatively higher than other types of incandescent lamps.

Following that, white and dark reference spectra were gathered for the experiment. The dark reference spectrum (FD) was obtained by turning off the light source and utilizing an opaque hood. Meanwhile, the white reference spectrum (FW) was obtained by measuring the raw data of deionized (DI) water. The collected spectra of the milk samples were designated as SP_1. The calibrated original absorbance spectra, TC, were computed utilizing Eq. (1):

1 $${\text{Absorbance}}\;\left( {\text{A}} \right) = - \log {\text{T}} = \log {{\mathbf{1}} \mathord{\left/ {\vphantom {{\mathbf{1}} {\varvec{T}}}} \right. \kern-0pt} {\varvec{T}}}.$$

The absorbance spectra were then calibrated to 100% transmission (T) with distilled water at the maximum wavelength (λ_max_). The calibrated spectra (TC) were used in further analyses, such as spectral preprocessing, data dimension reduction, and modeling.

### Processing of spectra and preparation of stains

Regarding the fluid apparatus testing process for spectral analysis, three experiments were designed to confirm the analytical capability of the proposed microfluidic device.

Experiment 1 tested a standard device (cuvette) against a microfluidic device to demonstrate that the values of the sample can be measured using spectroscopic analysis. Three testing modes were conducted, which included absorbance, transmittance, and fluorescence modes. The samples used for testing were a standard solution of Potassium Permanganate for absorbance and transmittance modes, and a 0.01 M Fluorescein solution for fluorescence mode. The Fluorescein solution was able to emit light in the wavelength range of 518–520 nm.

Experiment 2 was conducted to test the microfluidic device’s ability to analyze milk using spectroscopy. The experiment was divided into several tests, including light absorption testing and light transmission test comparing milk to Potassium Permanganate, and fluorescence test using Fluorescein, AO, and mixed AO-milk samples. Additionally, the study employed a cuvette to compare the fluorescence test analysis results between the microfluidic device and the cuvette and to evaluate the milk analysis abilities of the microfluidic device.

Experiment 3 was conducted to test various types of milk samples using a microfluidic device and to compare the results with the measurements obtained from the Fossomatic. The purpose of the experiment was to confirm whether the microfluidic device was capable of analyzing differences in milk samples as effectively as the Fossomatic, which served as the reference in Table 1.

#### Preparation of stains

The milk samples were tested by dyeing them with AO at a ratio of 1:10. AO has an excitation maximum wavelength at 502 nm and an emission maximum wavelength at 525 nm, producing a green fluorescence. AO is a nucleic acid-binding dye that can permeate cells and bind to DNA or RNA, emitting red fluorescence. This unique property makes AO useful for studying the cell cycle, as it can interact with DNA or RNA through interference or electrostatic attraction. The binding process is similar for DNA and RNA, resulting in a similar fluorescence spectrum. As a result, AO can be used to image primary lysosomes and phagolysosomes, which may contain its products. For apoptotic cell phagocytosis, dyeing is commonly performed under an epifluorescence microscope and FCM. AO stains are particularly useful for the rapid screening of routinely sterilized samples, and therefore are recommended for the microscopic detection of fluorescent microorganisms in direct smears prepared from clinical and non-clinical materials. Staining should be performed at an acidic pH of 5.5 to achieve a different staining effect for bacteria, resulting in orange stains, while tissue components will appear yellow to green.

### Microfluidic device fabrication

#### UV-direct lithography

**Figure 3:**
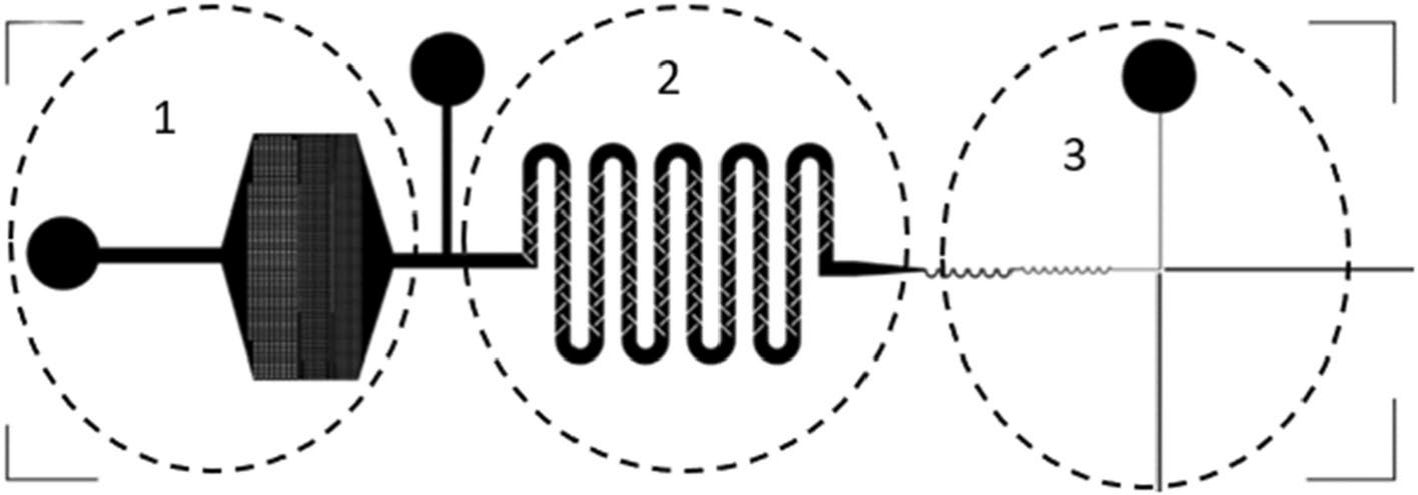
Structure of the microchannel for sorting and counting somatic cells (three-in-one structure). Structure 1 is the Somatic Cells Filter System, which filters out lymphocytes with a size of 7–18 µm and neutrophils with a size of 10–15 µm, accounting for 70–95% of total somatic cells in milk^26^. Structure 2 is the Mixing System, which mixes the somatic cells filtered from Structure 1 with AO dye. AO is typically used to stain the somatic cell membrane, which stains the DNA when viewed under a microscope. The fluorescence emitted at the emission maximum was 525 nm (green). This system does not require an incubation time of up to 30 min for the dye to adhere to the DNA. The mixing is done using a mixing scheme combined with proper flow control. The resulting mixture of dye and somatic cells is then analyzed using a third structure. The third structure arranges the somatic cell shells to form a single shell, which is analyzed using a Mini-Spectrometer for spectral reading using fluorescence principles. A fiber optics cabling structure with a 90° angle is designed to read fluorescence values, allowing for the analysis of the differences between each type of milk.

**Figure 4:**
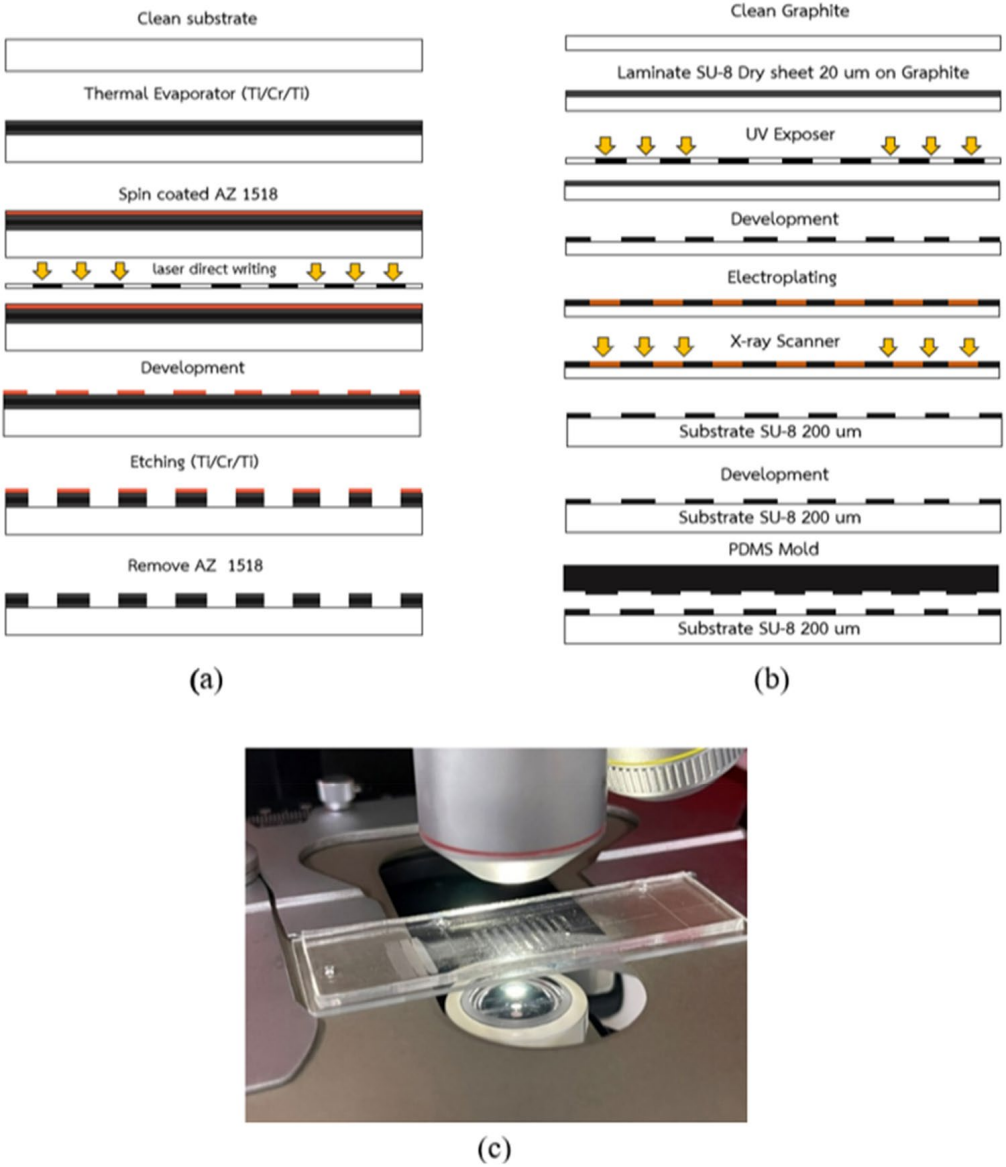
The fabrication process for microfluidic chips used in sorting and detecting somatic cells. (**a**) A Cr mask is fabricated using laser direct writing lithography. (**b**) An X-ray mask is created on a graphite substrate and then exposed through X-ray lithography on a 200 µm thick SU-8 photoresist. (**c**) The microchannel pattern is applied to the replicated PDMS to create the microfluidic chip.

The microfluidic chip design was created using Layout Editor^@^. The microchannel was developed as a three-in-one structure for filtering, mixing, and detecting lymphocytes in raw milk from cows, as shown in Fig. 3. Lymphocyte cells ranged from 7 to 18 µm, constituting 40% of the raw milk. Lymphocytes were filtered through the structure's filter and then mixed with AO staining, which binds to DNA/RNA in somatic cells. Staining was performed at an acidic pH to achieve differential staining, with bacteria showing orange stains and tissue components showing yellow to green stains. Lymphocytes were then detected in a single-cell structure (Structure 3) using a mini-spectrometer. Structure 1 had three layers that filtered lymphocytes of sizes 100, 50, and 20 µm. The entire microfluidic chip was fabricated using photolithography and was placed on a glass wafer using X-ray and soft lithographies. The microfabrication process is illustrated in Fig. 4. To create the X-ray mask, UV-laser direct writing lithography was employed. A patterned Cr mask was deposited using Ti/Cr/Ti on the glass substrate, and AZ P1518 photoresist was spin-coated on this substrate. The UV-direct written pattern on the substrate was then exposed to a UV dose of approximately 68 mJ/cm^2^. The glass substrate was developed around the patterned Cr mask, and Ti/Cr/Ti was etched. The AZ P1518 photoresist was removed using an acetone solution, and the patterned Cr mask appeared on the substrate. The X-ray mask was fabricated on a graphite substrate, which was laminate-coated using 20 µm-thick SU-8 dry sheets exposed to a UV dose of 50 mJ/cm^2^. The graphite substrate was patterned for the Cr mask and electroplated for the coating of an Au film on the patterned graphite substrate. The X-ray mask Au-patterned graphite had a thickness of approximately 20 µm for undergoing X-ray lithography. X-ray exposure was performed on the SU-8 sheets at a minimum dose of 100,000 mJ/cm^3^ to achieve a thickness of 200 µm for the sheets. To provide inlet and outlet fluidic interconnections, a replicated polydimethylsiloxane Sylgard 184 (PDMS-184) was used as the master mold replication. This PDMS mold had a microchannel pattern underneath, and the patterns were bonded using the plasma O_2_ method. The microdevice was attached to the optical fiber and connected using the mini-spectrometry method to set up the microchannel connection for the experiment.

**Figure 5:**
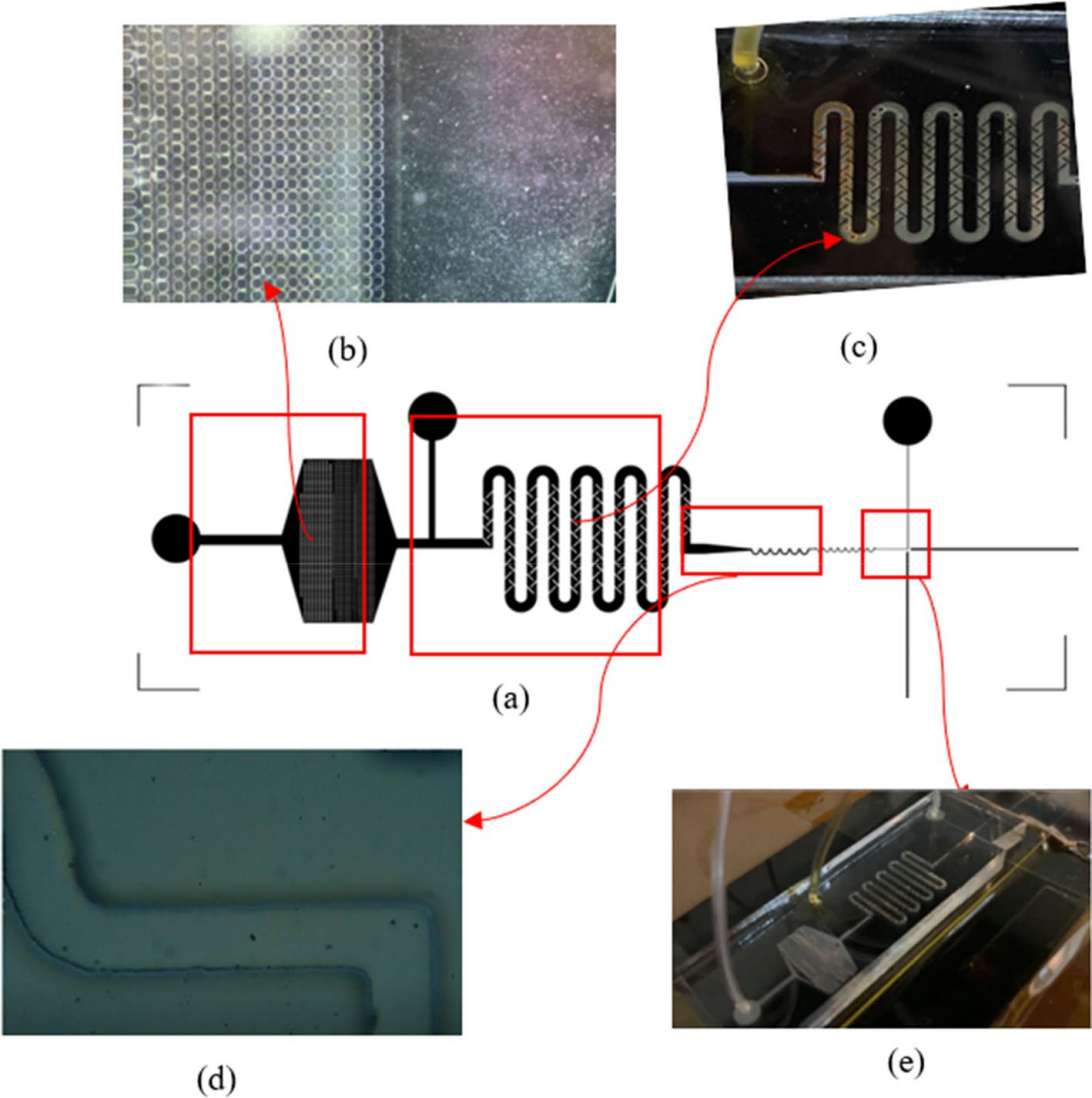
Microfluidic device for somatic cell classification. (**a**) Microfluidic device (**b**) somatic cells filter (**c**) somatic cells mixer (**d**) alinement of somatic cells to detection area (**e**) Detection area.

The microfluidic device was connected to the mini-spectrometer to measure the absorbance, transmittance, and fluorescence of the standard substance in order to determine the accuracy of the microfluidic device compared to the standard device (Cuvette) (shown in Fig. 2c,d). Raw milk and AO dye were controlled to flow into the device at a rate of 45 µL/min and 15 µL/min, respectively, as shown in Fig. 5. The BPS101 lamp was adjusted to achieve an intensity of 35,000. Sample analyses times of 100 ms and 300 ms were used for the absorbance, transmittance, and fluorescence mode, respectively.

## Results and discussion

**Figure 6:**
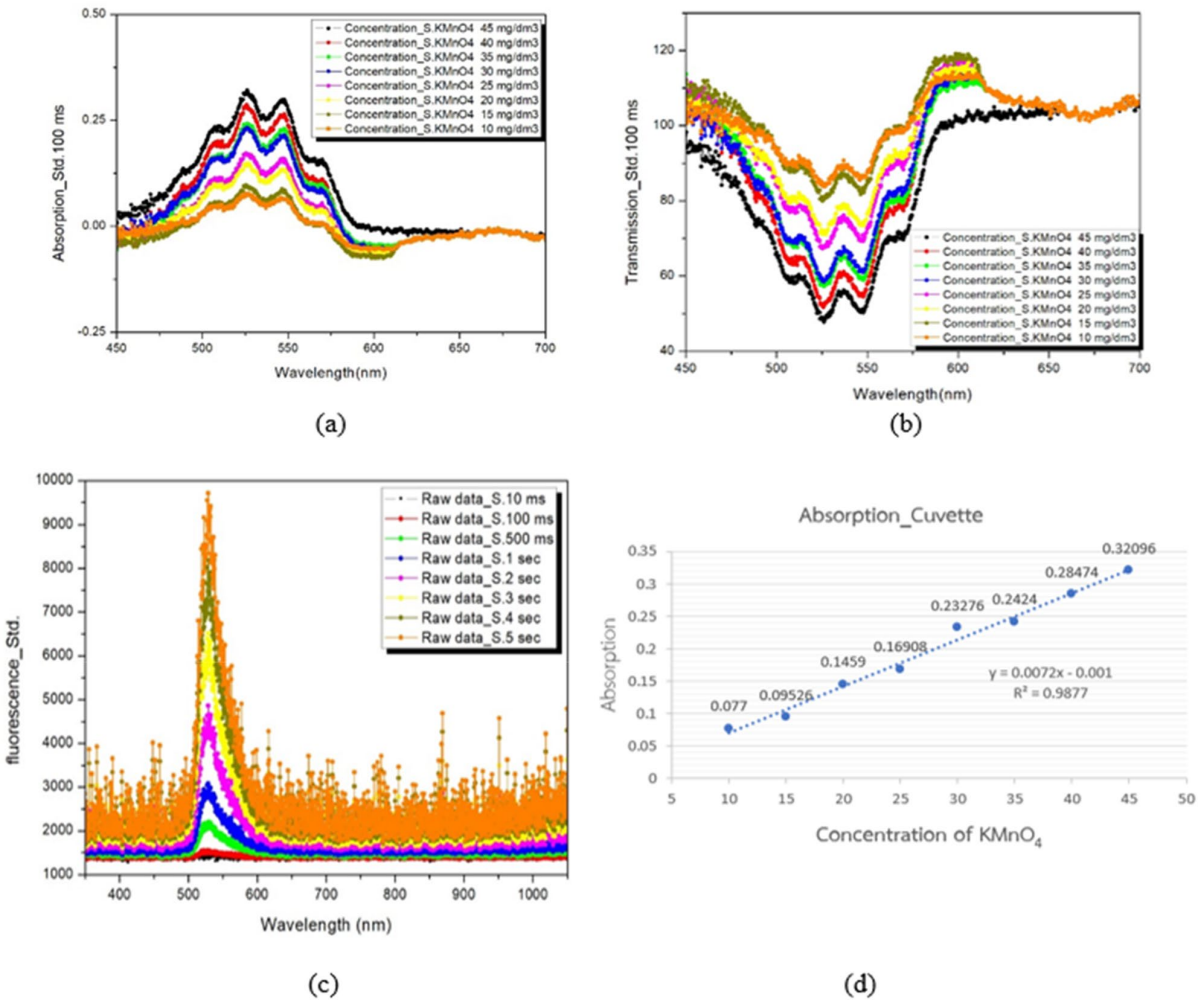
The results of testing potassium permanganate (KMnO_4_) concentration in the cuvette. (**a**) Absorbance mode (**b**) transmittance mode and (**c**) 0.01 M Fluorescein for Fluorescence mode (**d**) Analytical accuracy of the cuvette for potassium permanganate in absorption mode.

**Figure 7:**
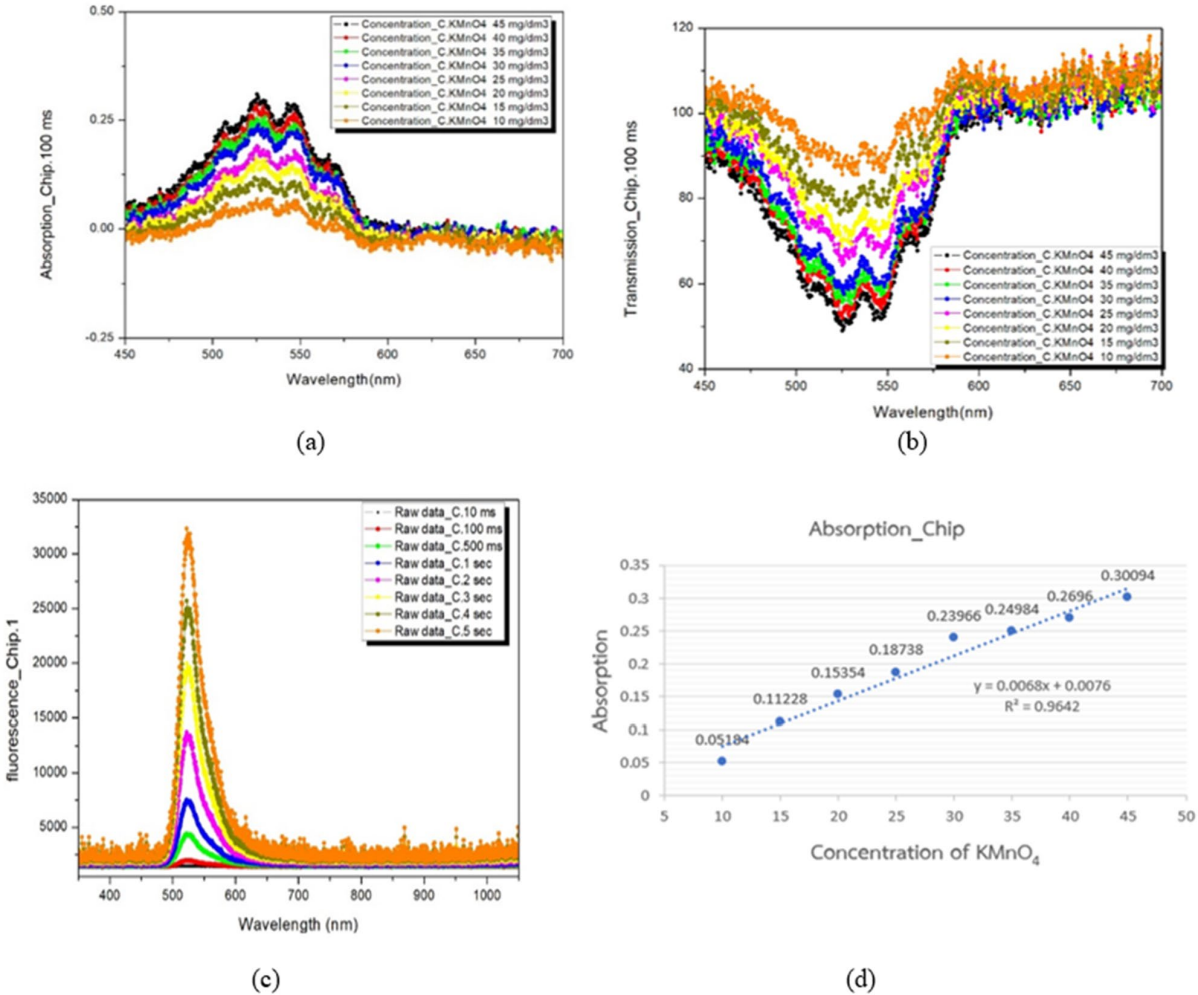
The results of testing potassium permanganate (KMnO_4_) concentration in the microfluidic device. (**a**) Absorbance mode (**b**) transmittance mode and (**c**) the 0.01 M Fluorescein for Fluorescence mode (**d**) Analytical accuracy of a microfluidic chip for potassium permanganate in absorption mode.

The quality of milk was measured using a photospectrometer, which worked on the principle of light. Two types of equipment— the standard equipment (Cuvette) and the proposed microfluidic device—were used as shown in Figs. 6 and 7, and their measurement accuracies were compared.

The photo-spectrometer optical technique was used to examine the milk quality results. This technique analyzes a solution's absorbance, transmittance, and fluorescence, which can determine its properties. The test was carried out using potassium permanganate as standard substance for absorbance and transmittance mode to demonstrate the functionality of the cuvette and the microfluidic chip, with the results shown in Figs. 6a,b and 7a,b, respectively. Potassium permanganate, which has a spectral maximum wavelength of 525 nm, was used to determine the device’s ability to perform an analysis in the absorbance and transmittance modes. In fluorescence mode, the test was carried out using a standard substance 0.01 M Fluorescein to demonstrate the functionality of the cuvette and the microfluidic chip, with the results shown in Figs. 6c and 7c. Fluorescein, which has a spectral maximum wavelength of 518 nm, was used to determine the device’s ability to perform an analysis in fluorescence mode.

In addition, we conducted an analysis to assess the accuracy of the microfluidic device in absorbance mode compared to the cuvette, as illustrated in Figs. 6d and 7d. The results indicated that the microfluidic device exhibited a reliable r-squared value of 0.9642, while the cuvette had an r-squared value of 0.9877, with a variance difference of only 0.0235. This experiment shows that the microfluidic device can be tested using spectroscopy and provide accuracy that is comparable to that of the standard cuvette holder.

**Figure 8:**
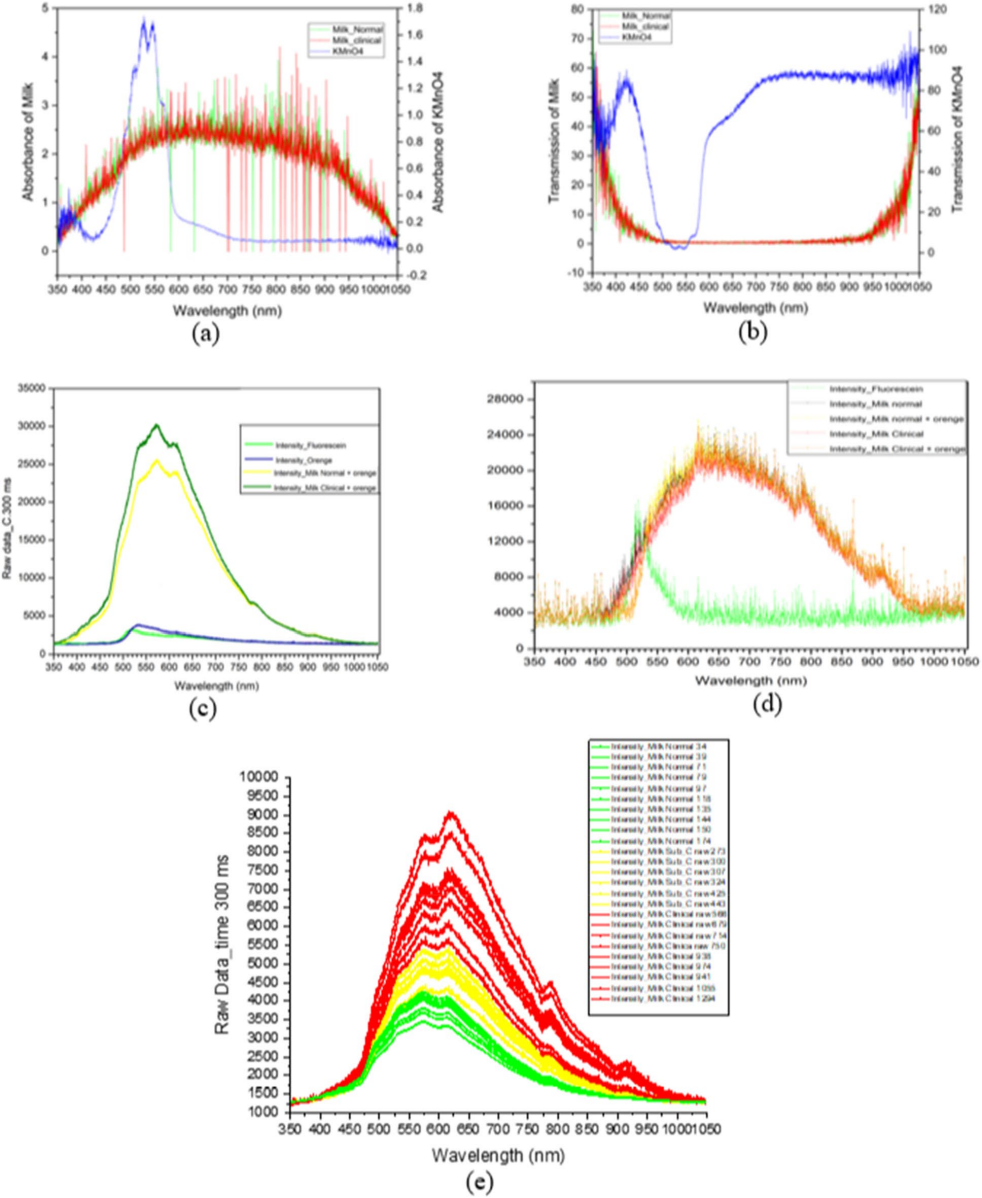
The testing milk and KMnO_4_ in the microfluidic chip (**a**) absorbance mode and (**b**) transmittance mode. (**c**) Testing 0.01 M Fluorescein, AO, and a mix of milk and AO in microfluidic chip fluorescence mode. (**d**) Testing 0.01 M Fluorescein, AO, and a mix of milk and AO in the standard equipment (cuvette) (**e**) comparison of accuracy under microfluidic device and Fossomatic cell counting instrument. The red line shows the clinical milk, the yellow line shows the sub-clinical milk, and the green line shows the normal milk.

The subsequent experiment involved testing milk in a microfluidic chip, with tests conducted in absorption, transmission, and fluorescence modes. The absorption and transmission modes were tested using a standard solution of potassium permanganate compared to milk, and the test results are presented in Fig. 8a and b. It was found that the cuvette testing was unable to measure milk through absorption and transmission modes, although such modes were possible for potassium permanganate. Meanwhile, the fluorescence mode was tested using Fluorescein, OA, and milk mixed with AO; the test results are presented in Fig. 8c, which indicates that the microfluidic device is capable of detecting differences in milk. Additionally, it was compared against the cuvette, and the results are presented in Fig. 8d, showing that the cuvette was unable to analyze the differences. This experiment thus demonstrates that the microfluidic chip can analyze milk in fluorescence mode.

To evaluate the performance of the microfluidic device in comparison to the CMT and Fossomatic reference results, a final test was conducted. The microfluidic device designed for raw milk was analyzed using a mini-photospectrometer. For this experiment, somatic measurements were assumed to be cell-level measurements. The CMT method and the regular method for analyzing abnormal raw milk both demonstrated the presence of somatic cells in the milk. The CMT reagent reacted with the raw milk, leading to the breakdown of hematopoietic cells, resulting in thick and viscous milk. The Fossomatic instrument employed the FCM measurement principle, which utilized a flow and dye mixing system to provide cell-by-cell metering. To optically measure cell-level attributes, measurements from the cuvette had to be scaled down to a size closer to that of the somatic cells. To accomplish this, a microfluidic system was designed to transport a group of cells into a beam inspection area similar in size to that of the cells. Finally, the somatic cells were transported to the analysis area, where the results were analyzed using a mini-spectrometer. The milk analysis was designed for the light absorbance, transmittance, and fluorescence modes. Figure 8e displays the analysis results, which were compared to the accuracy of the analysis performed with the Fossomatic machine, yielding an accuracy of up to 95%, as shown in Table 2.

**Table 2:** Number of errors under measurement of SCC in milk using the CMT method and microfluidic chip compared to the measurement standard.

Sample	Measurement accuracy with CMT	Measurement accuracy with microfluidic chip	Fossomatic
Normal milk (40 samples)	50	38	40
Sub-clinical (40 samples)	35	42	40
Clinical (20 samples)	15	20	20
Accuracy (%)	**74**	**95**	**100**

However, the components of the designed chip still had some disadvantages. Although the microfilter could filter somatic cells that were less than 50 µm in size and only allow these cells to pass through the mixing section, larger particles that were blocked at the beginning decreased the flow rate and might have caused an imbalance in the concentration between the sample and the dye. The initially blocked large particles can reduce the flow rate and result in an uneven concentration between the sample and the dye. Moreover, the blockage causes the microfluid device to become clogged after 10 min, which means that it can only be used for less than 10 min. The researchers plan to overcome these disadvantages by designing a removal system for somatic cells larger than 50 µm to avoid the aforementioned problems.

## Conclusions

An analytical microfluidic device has been designed and fabricated, utilizing the principle of a mini-spectrometer to detect high somatic cell count in milk. The microfluidic device can analyze abnormalities in the fluorescence modes, thereby characterizing milk infections through different spectral curves. The fluorescence magnitude of the infected milk with high somatic counts was higher than that of normal milk without somatic cells. The experimental results of the microfluidic device were consistent with those of the standard Fossomatic device, verifying the accuracy of the measurements with up to 95% precision. Therefore, the microfluidic device could detect mastitis trends in cows comparable to those of Fossomatic device. These results may be utilized in creating a prototype device capable of detecting the presence of mastitis within the spectrum.

## Data Availability

The datasets generated and/or analyzed during the current study are not publicly available due to the impact on cow milk industrials in our country but are available from the corresponding author upon reasonable request.
